# Catastrophic Influence of Global Distortional Modes on the Post-Buckling Behavior of Opened Columns

**DOI:** 10.3390/ma13153314

**Published:** 2020-07-25

**Authors:** Andrzej Teter, Zbigniew Kolakowski

**Affiliations:** 1 Department of Applied Mechanics, Faculty of Mechanical Engineering, Lublin University of Technology (LUT), Nadbystrzycka 36, PL–20-618 Lublin, Poland; a.teter@pollub.pl; 2 Department of Strength of Materials, Faculty of Mechanical Engineering, Lodz University of Technology, Stefanowskiego 1/15, PL-90-924 Lodz, Poland

**Keywords:** C-section, TH-section, distortional mode, medium length, interactive buckling, compression, Koiter’s theory, FEM

## Abstract

The multimodal buckling of thin-walled isotropic columns with open cross-sections under uniform compression is discussed. Column lengths were selected to enable strong interactions between selected eigenmodes. In the case of short columns or very long ones subjected to compression, single-mode buckling can be observed only and the effect under discussion does not occur. In the present study, the influence of higher global modes on the load-carrying capacity and behavior in the post-buckling state of thin-walled structures with open cross-sections is analyzed in detail. In the literature known to the authors, higher global modes are always neglected practically in the analysis due to their very high values of bifurcation loads. However, the phenomenon of an unexpected loss in the load-carrying capacity of opened columns can be observed in the experimental investigations. It might be explained using multimode buckling when the higher global distortional-flexural buckling modes are taken into account. In the conducted numerical simulations, a significant influence of higher global distortional-flexural buckling modes on the post-buckling equilibrium path of uniformly compressed columns with C- and TH-shaped (the so-called “top-hat”) cross-sections was observed. The columns of two lengths, for which strong interactions between selected eigenmodes were seen, were subject to consideration. Two numerical methods were applied, namely, the semi-analytical method (SAM) using Koiter’s perturbation approach and the finite element method (FEM), to solve the problem. The SAM results showed that the third mode had a considerable impact on the load-carrying capacity, whereas the FEM results confirmed a catastrophic effect of the modes on the behavior of the structures under analysis, which led to a lack of convergence of numerical calculations despite an application of the Riks algorithm. All elastic-plastic effects were neglected.

## 1. Introduction

Cold-formed isotropic columns under compression are commonly used as load-carrying elements in structures of any type. Their behavior under increasing loading depends on numerous factors, such as geometrical dimensions, kind of load, initial imperfections, and material properties. In the majority of cases, the performance of thin-walled structures results not from their strength but predominantly from their stability. In the general case, different buckling modes occur. Short columns and very long ones are subject to single-mode buckling and it is difficult to observe the effects of interactions of various buckling modes. These are local and global buckling modes, respectively. Only for medium-long columns, multimodal buckling could be observed. The key problem consists of a selection of eigenmodes, which are accounted for in the multimodal analysis. In the literature, it is easy to find many monographs, e.g., [1,2], and papers, e.g., [3,4,5], presenting the state of the art in the field of the nonlinear stability of thin-walled structures. Despite this fact, these issues have not been well recognized so far and are the subject of interest for numerous researchers. Numerical methods and theories, which are applied in the nonlinear analysis of stability and load-carrying capacity, have been constantly developed and improved. Among them, one can mention those widely used, namely: The Generalized Beam Theory (GBT), DSM (Direct Strength Method), and its modifications, e.g., the GBTUL (Generalized Beam Theory University of Lisbon), FSM (Finite Strip Method), FEM (Finite Element Method), and cFEM (constrained Finite Element Method). We focus only on recent publications devoted to the interactive buckling of thin-walled beams with open cross-sections by Ádány and Schafer [6,7,8,9], Davison [10], Hancock [11], Rasmussen [12,13], Silvestre [14], Szymczak [15], and Camotim et al. [16,17,18,19,20,21,22,23,24,25,26], and many more can be enumerated.

Development of the theory of the interactive buckling of thin-walled structures was discussed in the survey paper [11]. Martins, Camotim, Gonçalves, and Dinis [20,21,22,23] investigated interactive buckling and its effect on the post-buckling behavior and failure in columns [20] and beams [21,22,23] of various cross-sections. In [21], it is noticed that “secondary global-bifurcation D-G interaction—SGI” or “secondary distortional-bifurcation D-G interaction SDI” can occur when the beam geometry has an initial imperfection corresponding to three buckling modes (one distortional and two global).

The phenomenon of an unexpected loss in the load-carrying capacity of C-beams under bending was observed in the experimental investigations presented in [27,28]. In [28], an unexpected behavior of the holders used to support and load the models, which consisted of a rotation of the holders from their plane of bending, was observed. That behavior was interpreted as an effect of the secondary global distortional-lateral buckling mode on the interactive buckling of the C-section under bending, in the web plane. The results of further theoretical investigations were published in [29,30]. In [30], it is analyzed at what length of the steel C-beam under bending the effect of the second global mode on the load-carrying capacity was most considerable. The conclusions coming from those analyses pointed to beams of medium length. In [28,29,30], the problem was solved with the semi-analytical method (SAM) developed by the authors and based on Koiter’s asymptotic theory [31,32]. In the SAM, all analyzed buckling modes corresponding to bifurcation loads and the number of halfwaves along the longitudinal direction are calculated based on equilibrium equations and boundary conditions with the modified numerically strict transfer matrix method. This method is very useful for the interpretation of interactions of various buckling modes in the full range of loading. Moreover, it facilitates the understanding of the phenomena that occur during coupled buckling. In [33,34], this subject was further analyzed but extended to composite beams. Additionally, FEM analyses were conducted to validate the proposed SAM model and to verify the post-buckling equilibrium paths and load-carrying capacity attained. Such a comparison enables the scope to which the method can be applied to be determined, when the secondary global mode should be considered regarding the secondary global buckling mode and the range of beam lengths. The examples of computations cover the post-buckling equilibrium paths of C- and top-hat (TH)-section beams with given lengths, made of steel and laminates with different arrangements of plies.

The present paper is a continuation and expansion of the above-mentioned investigations for steel C- and TH-section columns with medium length and under uniform compression. The phenomenon of an unexpected loss in the load-carrying capacity of opened columns can be observed in the experimental investigations. It is worth mentioning that higher buckling loads are huge, but considering it in SAM leads to the significant decrease in the post-buckling equilibrium path and load-carrying capacity. It might be explained using multimode buckling when the higher global distortional-flexural buckling modes are taken into account. The results of computations were obtained with two numerical methods, the SAM and the FEM, where the second one was used for verification purposes. The considerations were limited to isotropic structures due to easier interpretations of the results than for composite structures, including FGMs and so on [35,36].

## 2. Formulation of the Problem

**Figure 1:**
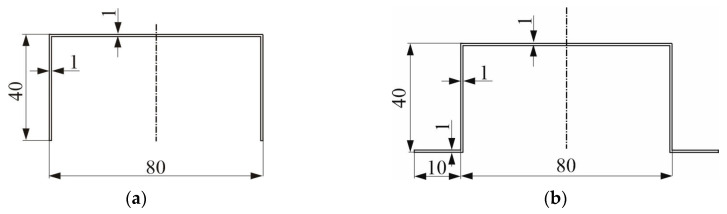
Outlines of the cross-sections and their dimensions: C-section (**a**) and top-hat (TH)-section (**b**).

The multimodal elastic buckling of C-section (Figure 1a) and TH-section (the so-called “top-hat”) (Figure 1b) columns with medium lengths and under compression, made of isotropic materials like steel, was analyzed. The material the columns were made of satisfies Hooke’s law. The column lengths were chosen to ensure strong interactions between the selected buckling eigenmodes. The numerical simulations were conducted with two methods, namely the semi-analytical method (SAM) and the finite element method (FEM), to discuss the effect of global distortional-flexural modes on the post-buckling behavior of the columns under compression in detail.

### 2.1. Semi-Analytical Method (SAM)

The analytical-numerical method (ANM) [31] and the semi-analytical method (SAM) [32,37] based on Koiter’s asymptotical perturbation theory within Byskov–Hutchinson’s formulation had been developed for more than 30 years by the authors [28,29,30,33,34]. The methods allow one to solve the nonlinear stability problem of thin-walled structures made of isotropic, orthotropic, and composite materials. The structures under consideration are prismatic thin-walled columns built of plates connected along longitudinal edges and subjected to complex longitudinal loading. To account for all possible modes of global, local, and coupled buckling, a plate model (i.e., 2D) of thin-walled structures is applied. The columns are freely supported at their ends. For each plate, the exact geometrical relationships (i.e., full Green’s strain tensor) are assumed:

(1) $$\begin{array}{l} \varepsilon_{x}^{}=u_{,x}+\frac{1}{2}(w_{,x}^{2}+v_{,x}^{2}+u_{,x}^{2})\\ \varepsilon_{y}^{}=v_{,y}+\frac{1}{2}(w_{,y}^{2}+u_{,y}^{2}+v_{,y}^{2})\\ 2\varepsilon_{xy}^{}=\gamma_{xy}^{}=u_{,y}+v_{,x}+w_{,x}w_{,y}+u_{,x}u_{,y}+v_{,x}v_{,y} \end{array}$$

and

(2) $$\kappa_{x}=-w_{,xx}\;\kappa_{y}=-w_{,yy}\;\kappa_{xy}=-2w_{,xy}{}_{}^{}$$

where *u, v, w*—components of the displacement vector of the plate along the *x-, y-,* and *z*-axis direction, respectively.

According to Koiter’s theory, the fields of displacement and the fields of sectional forces are distributed into a series with respect to the dimensionless deflection amplitude [31,32,37]:

(3) $$\begin{array}{l} U\equiv (u,v,w)=\lambda U_{0}+\zeta_{r}U_{r}+\zeta_{r}^{2}U_{rr}+\ldots \\ N\equiv (N_{x},N_{y},N_{xy})=\lambda N_{0}+\zeta_{r}N_{r}+\zeta_{r}^{2}N_{rr}+\ldots \end{array}$$

where λ—load factor, *U*_0_*, N*_0_—the pre-buckling (i.e., unbending) fields, *U_r_, N_r_*—the first-order nonlinear fields, *U_rr_, N_rr_*—the second-order nonlinear fields of the displacement and the sectional force, respectively. The range of indices is [1, *J*], where *J* is the number of interacting modes. It is assumed that the summation is over the repeated indices.

In thin-walled structures with the initial geometric imperfections *Ū* (only the linear initial geometric imperfections corresponding to the shape of the *r*-th buckling mode, i.e., *Ū* = ζ_r_^*^U_r_, are considered), the total potential energy can be described by the following formula:

(4) $$\Pi =-\frac{1}{2}M^{2}{\bar{a}}_{0}+\frac{1}{2}\sum_{r=1}^{J}{\bar{a}}_{r}\zeta_{r}^{2}\left(1-\frac{M}{M_{r}}\right)+\frac{1}{3}\sum_{p}^{J}\sum_{q}^{J}\sum_{r}^{J}{\bar{a}}_{pqr}\zeta_{p}\zeta_{q}\zeta_{r}+\frac{1}{4}\sum_{r}^{J}{\bar{b}}_{rrrr}\zeta_{r}^{3}-\sum_{r}^{J}\frac{M}{M_{r}}{\bar{a}}_{r}\zeta_{r}^{*}\zeta_{r}$$

and the equations of equilibrium corresponding to (4) are:

(5) $$\left(1-\frac{P}{P_{r}}\right)\;\zeta_{r}+a_{pqr}\zeta_{p}\zeta_{q}+b_{rrrr}\zeta_{r}^{3}=\frac{P}{P_{r}}\zeta_{r}^{*}\;r=1,\;\ldots ,\;J$$

where:

(6) $$a_{pqr}={\bar{a}}_{pqr}/{\bar{a}}_{r}\;b_{rrrr}={\bar{b}}_{rrrr}/{\bar{a}}_{r}$$

Expressions for coefficients: *a_o_*, *a_r_*, *a_pqr_*, *b_rrrr_* are calculated by known formulae [1,2,3,32,37]. The post-buckling coefficients *a_pqr_* depend only on the buckling modes, whereas the coefficients *b_rrrr_* also depend on the second-order field. The nonlinear stability problem of thin-walled structures in the first-order approximation of Koiter’s theory was solved with the modified analytical-numerical method (ANM) presented in [31]. In the ANM, an exact transfer matrix method is applied as opposed to the finite strip method. The ANM should be extended by the second-order approximation of the theory. The second-order coefficients are estimated with the semi-analytical method (SAM) [32,37]. In the SAM, it is postulated to define approximated values of the coefficients *b_rrrr_* based on the linear buckling problem. Such an approach allows for an exact determination of values of the coefficients *a*_pqr_, according to the nonlinear Byskov–Hutchinson’s theory.

The relative shortening of column ends Δ/Δ*_min_* is defined as a function of *P/P_min_* through differentiation of potential energy expression (4) with respect to *P/P*_min_ [28,29,30,31,32]:

(7) $$\frac{\Delta }{\Delta_{\min}}=\frac{P}{P_{\min}}\left[1+\frac{P_{\min}}{P{\bar{a}}_{0}}\sum_{r=1}^{J}\frac{P_{\min}}{P_{r}}{\bar{a}}_{r}\zeta_{r}\left(0.5\zeta_{r}+\zeta_{r}^{*}\right)\right]$$

where Δ*_min_*—minimal shortening of the column corresponding to the minimal value of the bifurcation load *P*_min_.

In the presented study, a three-modal approach was applied at most (i.e., *J =* 3 in the relationship (5)). This means that a model with a maximum of three degrees of freedom was assumed.

### 2.2. Finite Element Method (FEM)

To verify the SAM results, a commercial package Abaqus (Dassault Systèmes Simulia Corp., Johnston, RI, USA) [38] was applied in the finite element method (FEM) calculations. A numerical model of the thin-walled structures under analysis, with the boundary conditions adapted to the SAM, was developed. The columns under consideration were modeled with four-node shell elements with eight degrees of freedom in each node (element type: S8R). The element size equal to 2 mm was determined based on the previous experience of one of the authors and the analyses of convergence. In the FEM models of the thin-walled structures under analysis, the following was assumed: For the 300 mm-long C-channel, 12,000 elements and 96,640 degrees of freedom, whereas for the TH-channel, 13,500 elements and 108,720 degrees of freedom. In the case of longer C-channels, 20,000 elements and 160,640 degrees of freedom were assumed, while for longer TH-channel columns, 22,500 elements and 180,720 degrees of freedom were assumed. All simulations were conducted for whole thin-walled structures, because not only symmetrical modes can be discussed. In our case, symmetry boundary conditions can be applied. Both solutions are the same. Additionally, non-symmetrical buckling of the opened columns, which can follow from an interaction of anti-symmetrical modes, can be observed. They have been neglected in the present study.

**Figure 2:**
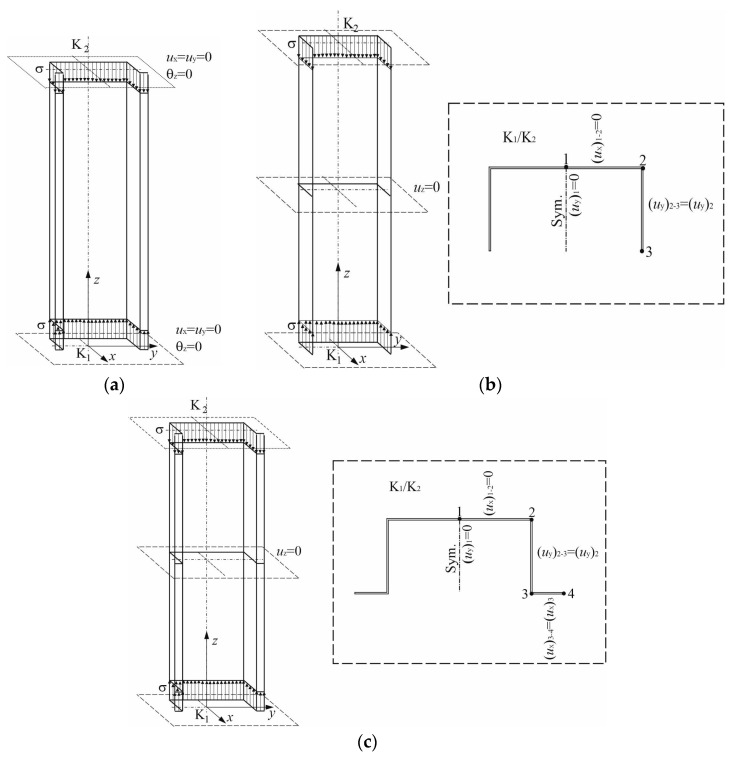
Boundary conditions: Classic variant of the column-free support (**a**) and modified boundary conditions for the C-channel (**b**) and the TH-channel (**c**) [39].

Moreover, the initial conditions corresponding to the free support of the column ends under uniform compression were modified. The assumption of classic boundary conditions (Figure 2a) in which the column ends (denoted as K1 and K2) are fixed in the cross-section plane of the column, that is to say: u_x_ = *u*_y_ = 0 and *ϑ_z_* = 0, and, additionally, at the end K1, the displacements are blocked along the length, that is to say: *u*_z_ = 0, leads to over-stiffening of the model. The determined eigenvalues for higher global buckling modes in the columns with open cross-sections under discussion were overestimated with respect to the results attained with the analytical-numerical method. To counteract it, the boundary conditions were modified as suggested in [39]. In this case, the boundary conditions are as follows (Figure 2b,c):

- In the middle of the column, displacements along the column length are equal to 0, i.e., *u*_z_ = 0,
- At both ends K1 and K2, it is assumed that the intersection point of the web with the cross-section axis (denoted as point 1 in Figure 2c) cannot displace along the *y*-axis, i.e., (*u*_y_*)*_1_ = 0. In segment 1–2, the web cannot displace along the *x*-axis, i.e., (*u*_x_*)*_1–2_ = 0. In segment 2–3, the arm displaces with respect to the *y*-axis identically as corner 2, i.e., *(u_y_)*_2–3_ *= (u_y_)*_2_. Reinforcement 3–4 displaces along the *x*-axis identically as corner 3, i.e., *(u_x_)*_3–4_ *= (u_x_)*_3_.

In Figure 2b, modified conditions for the C-column are presented, whereas those for the TH-column are to be found in Figure 2c.

Another variant of the modified boundary conditions was suggested by Szymczak and Kujawa [40,41,42]. Comparing the results of simulations of bifurcation loads for both the modified variants of the boundary conditions, one can state that they are consistent. The determined higher eigenvalues and post-buckling paths of equilibrium are identical in both cases of the modified boundary conditions. It is worth adding that the nonlinear stability problem was solved both with the Riks method and the Newton-Raphson method. The results did not differ; thus, in the present study, only the results for the Riks algorithm are presented. To attain numerical convergence, the computational timestep was highly diminished.

It should be mentioned that the cost/time ratio of the SAM calculations is more than two orders of magnitude lower than in the case of the FEM. Moreover, the SAM enables much easier analysis of the phenomena under investigation and their interpretation when compared to the FEM.

## 3. Results and Discussion

### 3.1. C-Channel Columns

In the first stage of the calculations, the stability of C-channel columns was investigated. The geometrical dimensions of the column cross-sections are presented in Figure 1, whereas their material constants were as follows: Young’s modulus—200 GPa and Poisson’s ratio—0.3. A linear-elastic model of the material and a column length equal to 300 and 500 mm were assumed. Solving the eigenproblem with both the methods (i.e., the SAM and the FEM), we determined eigenvalues and modes. The attained values of bifurcation loads are listed in Table 1. Additionally, the number of halfwaves *m*, which form on the column surface along the longitudinal direction, is given in brackets for the local modes. The following indices are used: 1—local buckling mode (when *m* > 1), 2—primary global buckling mode, 3—secondary global distortional-flexural mode, 4—third global distortional-flexural mode. The buckling modes where *m* = 1 are referred to as the global ones in the present paper.

Very good compatibility of the values of bifurcation loads (the so-called eigenvalues) from the SAM and the FEM was obtained owing to the modified boundary conditions assumed in the FEM. The determined values of local bifurcation forces are lower than the primary global values, as, for the column of the length of 300 mm, we have *P*_2_/*P*_1_ = 2.1, and for the length of 500 mm, *P*_2_/*P*_1_ = 4.5. The load ratios corresponding to higher modes are as follows: *P*_3_/*P*_1_ ≈ 15 and *P*_4_/*P*_1_ ≈ 70, and they have not been considered in the literature devoted to interactive buckling. The authors, despite this “impracticality”, deal with this problem as the main object in this study.

**Figure 3:**
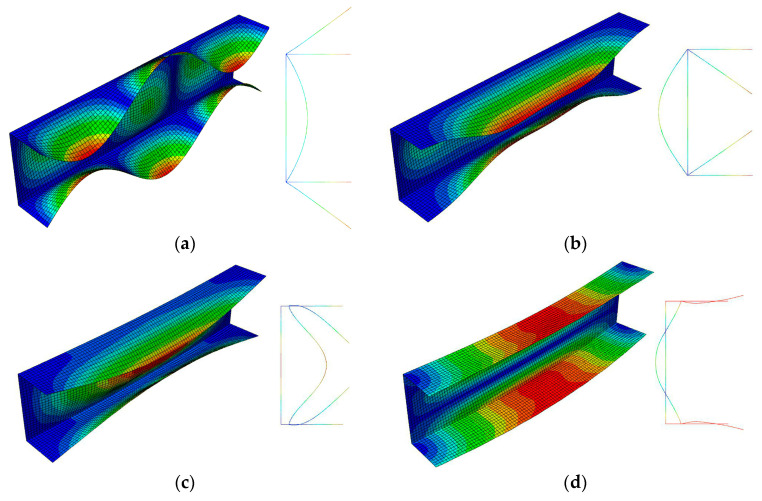
Buckling modes for the 300 mm-long C-section, FEM results. Subsequent modes are as follows: Local *m* > 1 (**a**), primary global (**b**), higher global distortional-flexural (**c**), and third global distortional-flexural (**d**).

**Figure 4:**
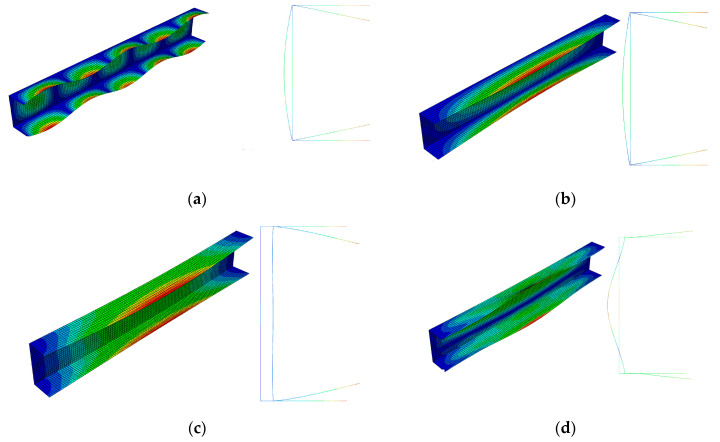
Buckling modes for the 500 mm-long C-section, FEM results. Subsequent modes are as follows: Local *m* > 1 (**a**), primary global (**b**), higher global distortional-flexural (**c**), and third global distortional-flexural (**d**).

The FEM buckling modes are presented in Figure 3 for the 300 mm-long column and in Figure 4 for the 500 mm-long column. The buckling mode, i.e., the eigenvector, is determined with an accuracy up to a certain constant C. This remark is necessary due to the buckling modes presented in Figure 3a (local mode *i =* 1) and in Figure 3b (primary global mode *i =* 2) for 300 mm. The amplitude of global buckling (Figure 3b) can be transformed through a change in the constant C into the constant −C, i.e., into the amplitude of the local mode in practice (Figure 3a) (cf. the shape of the mode amplitude at the right-hand side (Figure 3a,b)). Thus, these modes differ only in the number of halfwaves m along the longitudinal direction. Global modes for *i =* 3 (Figure 3c) and *i =* 4 (Figure 3d) are distortional-flexural modes, where, for the first of them, maximal displacements occur for the web, and for the second one, those occur for the plates.

**Figure 5:**
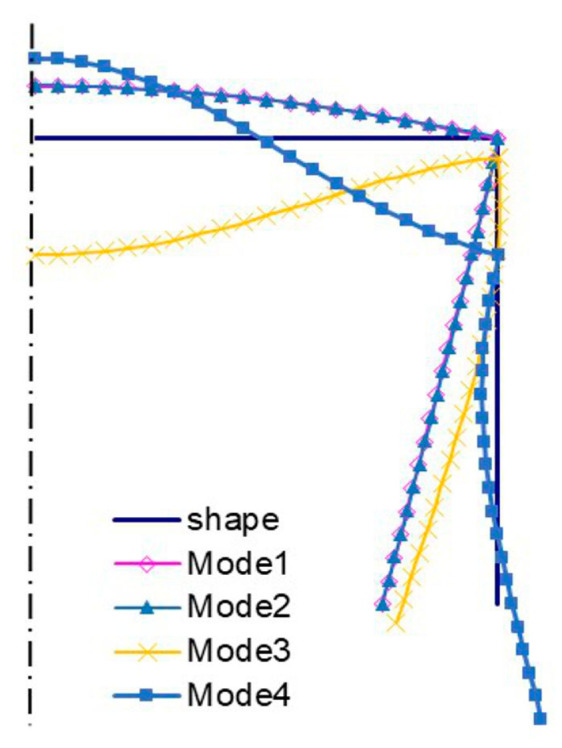
Buckling modes for the 300 mm-long C-section, SAM results. Subsequent modes are as follows: Local *m* > 1 (Mode1), primary global (Mode2), higher global distortional-flexural (Mode3), and third global distortional-flexural (Mode4).

**Figure 6:**
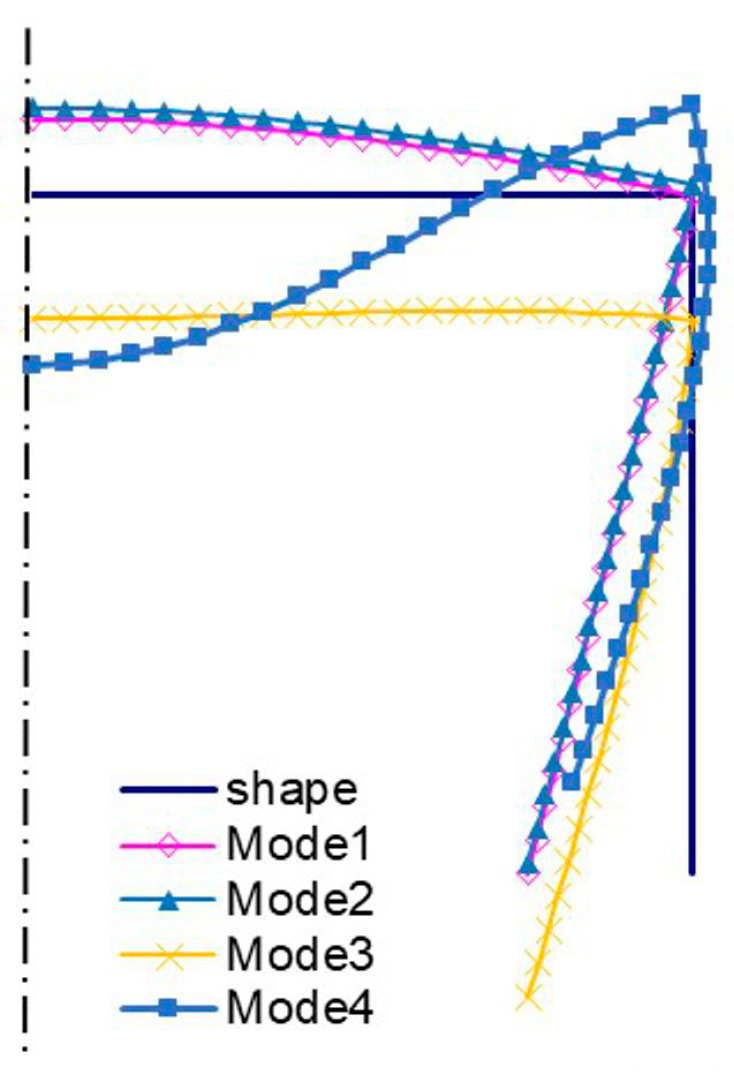
Buckling modes for the 500 mm-long C-section, SAM results. The same notation of modes as in Figure 5.

The amplitudes of the local buckling mode (*i =* 1, *m =* 5—Figure 4a) and the primary global mode (*i =* 2—Figure 4b) of the C-channel, which is 500 mm long, are the same, but they differ as far the number of halfwaves along the longitudinal direction is concerned. The secondary global mode (*i =* 3—Figure 4c) is the distortional-flexural mode, where the web displaces as for the “pure” flexural mode. The third global mode (*i =* 4—Figure 4d) is also the distortional-flexural one, but ‘simpler’ with respect to the third mode for 300 mm (Figure 3d). A similar statement can be made when the secondary global modes for 300 (Figure 3c) and 500 mm (Figure 4c) are compared. In Figure 5 and Figure 6, four buckling modes for 300 and 500 mm, obtained with the SAM, are shown, respectively. A very good agreement of the mode attained with the SAM and FEM for the given length (compare Figure 3 and Figure 5 to Figure 4 and Figure 6, correspondingly) can be seen.

The next stage consisted of an analysis of post-buckling equilibrium paths for the C-columns. Two-mode (*J =* 2 in Equation (5)) and three-mode (*J =* 3) approaches were considered for the SAM to facilitate an interpretation of the results. Moreover, the initial imperfections ζ_r_^*^ = |0.2|, where the signs were selected in the most disadvantageous way [30,31,32,34], were assumed.

**Figure 7:**
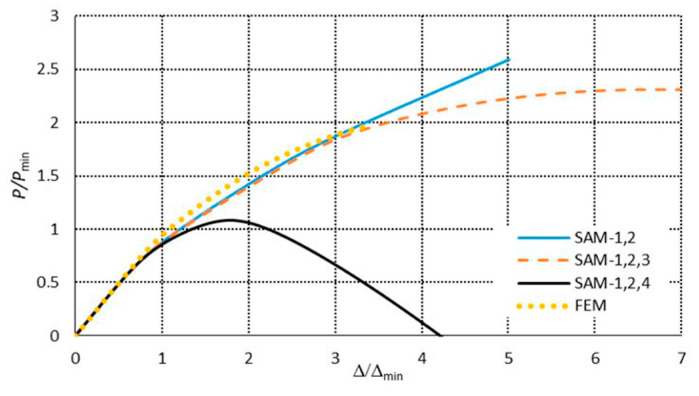
Post-buckling equilibrium paths in the dimensionless system for the 300 mm-long C-section.

**Figure 8:**
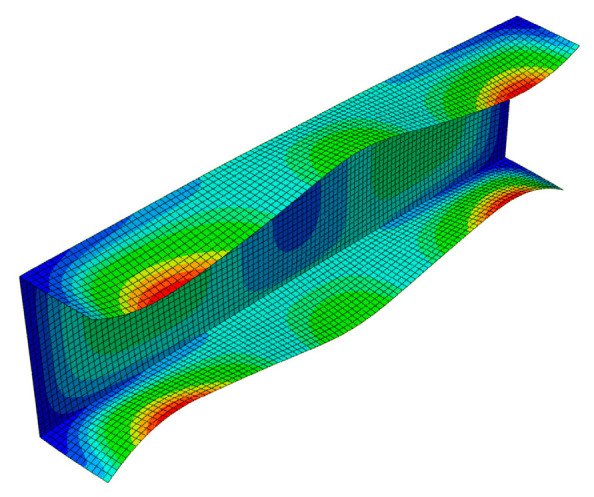
Mode of the 300-mm long C-section in the ultimate state, FEM results.

Figure 7 presents a collection of the SAM and FEM solutions showing post-buckling equilibrium paths in the system of the dimensionless load *P/P*_min_ = *P/P*_1_ as a function of the dimensionless shortening Δ/Δ*_min_ =* Δ/Δ_1_ (where *P*_min_ corresponds to the lowest value of the bifurcation load, and Δ*_min_* denotes the minimal shortening caused by the load *P*_min_) for the 300 mm-long column. The post-buckling paths for a coupled interaction of two modes (*J =* 2): The local mode (*i =* 1) with the primary global one (*i =* 2) (denoted here as SAM-1,2) and for two cases of interaction of three modes (*J =* 3): The local mode (*i =* 1) with the two lowest global modes (i.e., for *i =* 2,3) (denoted as SAM-1,2,3), and the local mode (*i =* 1) with the global primary mode (*i =* 2) and the third mode (*i =* 4) (denoted as SAM-1,2,4), are presented. For the two-mode approach, the post-buckling path grows monotonically. For the three-mode approach, the SAM-1,2,3 curve flattens, not reaching the ultimate load-carrying capacity, whereas, for the SAM-1,2,4, it attains the ultimate value of *P/P*_min_ ≈ 1.1 and then falls, which corresponds to an unstable equilibrium path. It follows from these comparisons that an effect of the third global mode (*i =* 4) is crucial for interactive buckling of the 300 mm-long C-column. An application of the SAM thus enables an easy analysis of the effect of the modes under study on post-buckling paths, which is not possible virtually with the FEM. The FEM was used to verify the SAM results. In the FEM calculations, the amplitude of the initial deflection equal to 0.2 of the component plate thickness was assumed for the local mode (*i =* 1) with the global primary mode (*i =* 2) and the second mode (*i =* 3) or third global mode (*i =* 4). The results were the same. A very good agreement between the FEM curve and the SAM-1,2,3 curve was obtained. The post-buckling curve SAM-1,2 begins to differ more and more from the FEM curve for the load *P/P*_min_ ≥ 1.8. It was not possible to verify the curve SAM-1,2,4 with the FEM. Attention should be paid to the FEM curve. It breaks catastrophically for *P/P*_min_ ≈ 1.95 and Δ/Δ*_min_*≈ 3.4 due to a lack of convergence of the Riks method. Thus, it is the ultimate value. None of the changes in the parameters of the Riks method allowed the calculations to continue. The authors put forward a hypothesis that when the secondary (*i =* 3) and third (*i =* 4) global modes begin to interact, numerical convergence of the FEM is lost, and the computations are catastrophically disrupted. This hypothesis requires further thorough investigations to be carried out. In Figure 8, a mode for the ultimate FEM value is shown. As can be easily seen in the central part of the C-channel, deflections of the plates and the web flattened out, with the symmetry of displacements remaining with respect to the cross-section axis.

**Figure 9:**
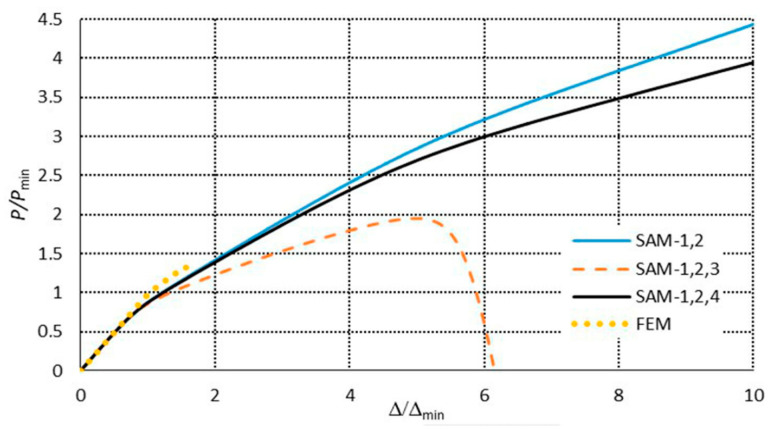
Post-buckling equilibrium paths in the dimensionless system for the 500 mm-long C-section.

**Figure 10:**
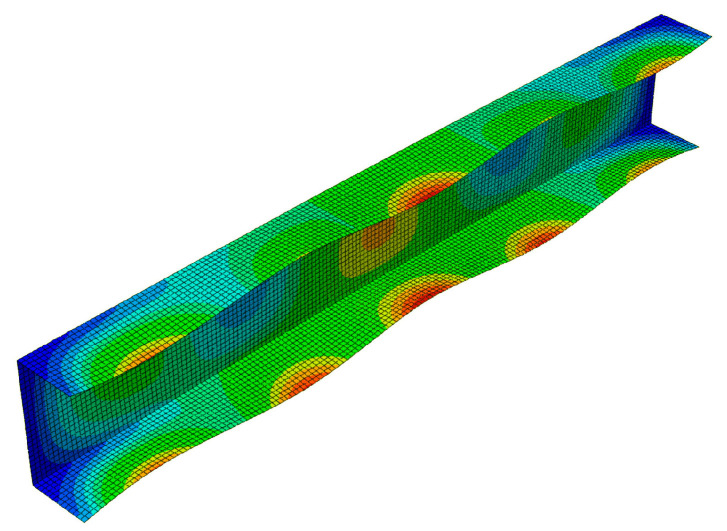
Mode of the 500 mm-long C-section in the ultimate state, FEM results.

In the next figures (Figure 9 and Figure 10), results for the 500 mm-long C-channel are presented. In Figure 9, the post-buckling equilibrium paths *P/P*_min_ = *P/P*_1_ as a function of the shortening Δ/Δ*_min_ =* Δ/Δ_1_ are shown for the SAM and the FEM. The curves SAM-1,2 for the two-mode approach (*J =* 2) and SAM-1,2,3 for the three-mode approach (*J =* 3) are situated close to each other up to the load *P/P*_min_ ≈ 2.5. The post-buckling curve SAM-1,2,4 differs from the remaining curves already at *P/P*_min_ > 0.85 and attains the ultimate load-carrying capacity for the load *P/P*_min_ ≈ 2 and the shortening Δ/Δ*_min_* ≈ 5.1. The FEM post-buckling equilibrium path is similar to the curves SAM-1,2 and SAM-1,2,3, but lies above them. In this case, we were not able to obtain a curve close to SAM-1,2,4 either. For example, for the 300 mm-long C-column, the FEM computations were disrupted catastrophically by a lack of numerical convergence for *P/P*_min_ ≈ 1.35 and Δ/Δ*_min_*≈ 1.65, which should be treated as the ultimate value. Figure 10 presents the buckling mode of the C-section at the ultimate point. Non-symmetrical buckling of C-section strips, which can follow from an interaction of anti-symmetrical modes, can be observed. They have been neglected in the present study.

For both the lengths of C-columns, a huge impact of high values of global buckling modes, which are unrealistic in the theory of interactive buckling from the practical point of view, can be seen. The present study is just an attempt to identify and to describe the phenomenon of the influence of secondary and third global distortional-flexural modes on the load-carrying capacity of medium-long C-channels.

### 3.2. TH-Channel Columns

In this section, the stability and post-buckling equilibrium paths of “top-hat”- section columns (Figure 1b) of the following lengths: 300 and 500 mm, will be discussed. Table 1 includes the bifurcation values of stresses obtained with the SAM and the FEM. Moreover, for the local mode (*i* = 1), the number of halfwaves m along the longitudinal direction is given in brackets. The same index notations are used as for the C-channels.

A very good agreement of the SAM and FEM results was obtained, similarly to the case of the C-channels. It should be noticed that an introduction of edge reinforcements in TH-section columns in comparison to C-section columns results is almost twice as high an increase in local bifurcational loads. What is more, the values of primary global loads for both lengths are very close. The ratio of the value of the second global mode to the local one is more than seven times higher, whereas for the third mode, it is 40 times higher, correspondingly.

**Figure 11:**
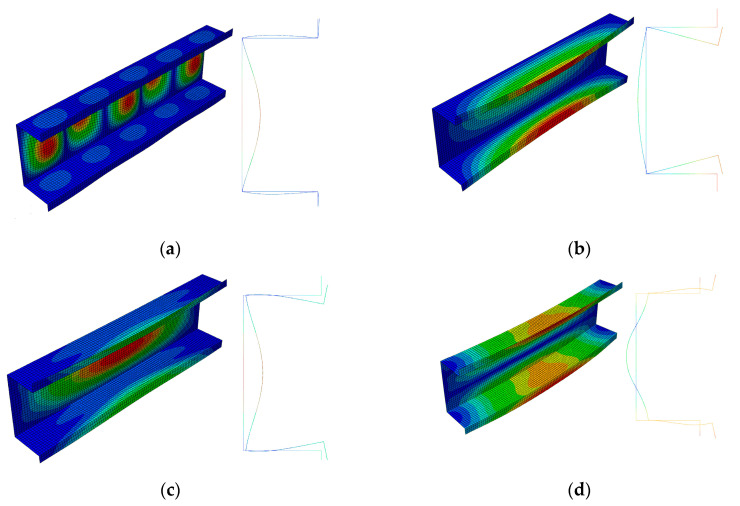
Buckling modes for the 300 mm-long TH-channel column, FEM results. Subsequent modes are as follows: Local *m* > 1 (**a**), primary global (**b**), higher global distortional-flexural (**c**), and third global distortional-flexural (**d**).

**Figure 12:**
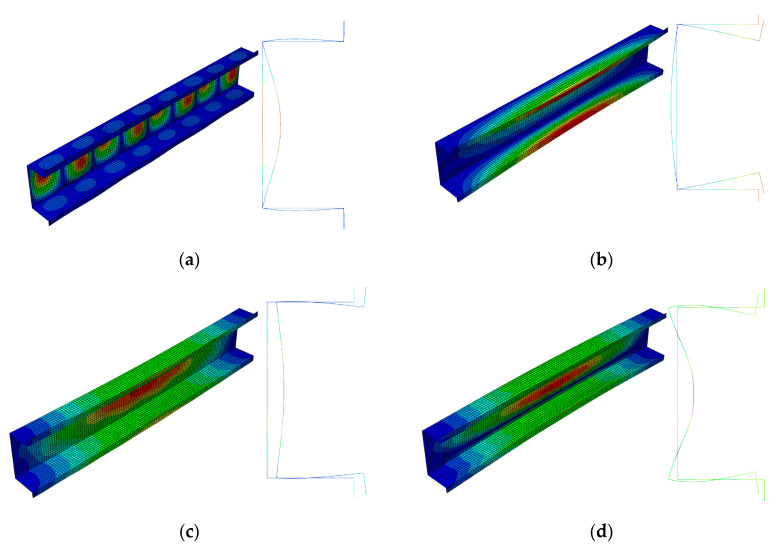
Buckling modes for the 500 mm-long TH-channel column, FEM results. Subsequent modes are as follows: Local *m* > 1 (**a**), primary global (**b**), higher global distortional-flexural (**c**), and third global distortional-flexural (**d**).

**Figure 13:**
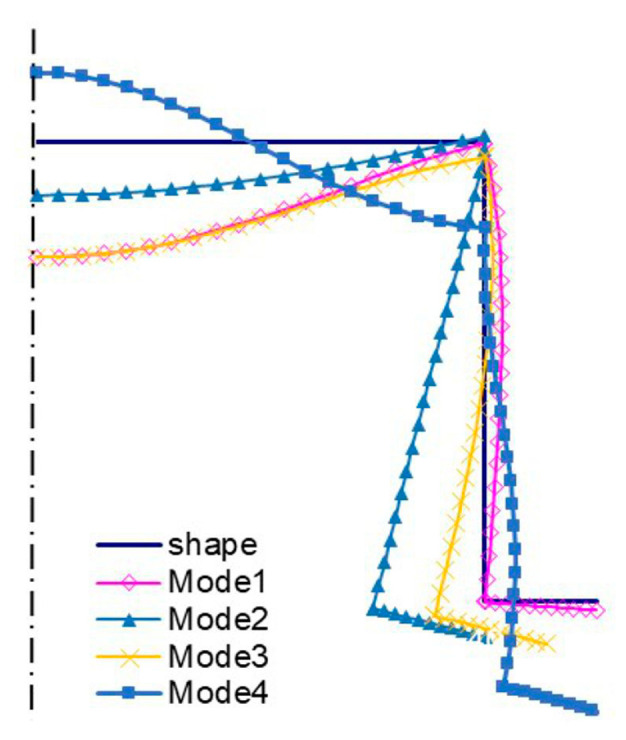
Buckling modes for the 300 mm-long TH-channel column, SAM results. The same notation of modes as in Figure 5.

**Figure 14:**
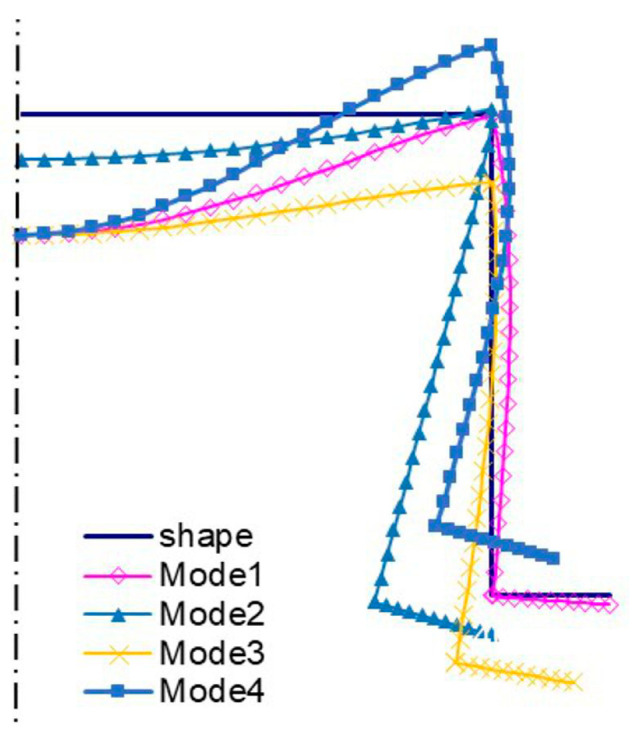
Buckling modes for the 500 mm-long TH-channel column, SAM results. The same notation of modes as in Figure 5.

Figure 11 shows bifurcation modes for the 300 mm column, whereas Figure 12 shows those for 500 mm. Local modes (Figure 11a and Figure 12a) are the ‘pure’ local ones, for which the edges do not displace. They differ in the number of halfwaves along the longitudinal direction. The amplitudes of modes (i.e., the shape of the mode in the cross-section) are identical and, analogously, primary global modes (*i =* 2) (Figure 11b and Figure 12b). In this case, the mode is distortional as the reinforced edge corner rotates together with the plate with respect to the web. Global secondary modes (*i =* 3) (Figure 11c and Figure 12c) are the distortional-flexural ones and they are similar, provided the interpretation for the C-channel from Figure 3a,b is assumed. The global secondary mode (*i =* 3) for the 500 mm column is even ”purer” than that for 300 mm, i.e., it is closer to the flexural one. Third modes (*i =* 4) are the distortional-flexural ones. In Figure 13 and Figure 14, buckling modes of columns 300 and 500 mm long, respectively, are presented for the SAM. When Figure 11, Figure 12, Figure 13 and Figure 14 are compared, it can be easily noticed that the buckling modes determined with the SAM and FEM agree very well.

**Figure 15:**
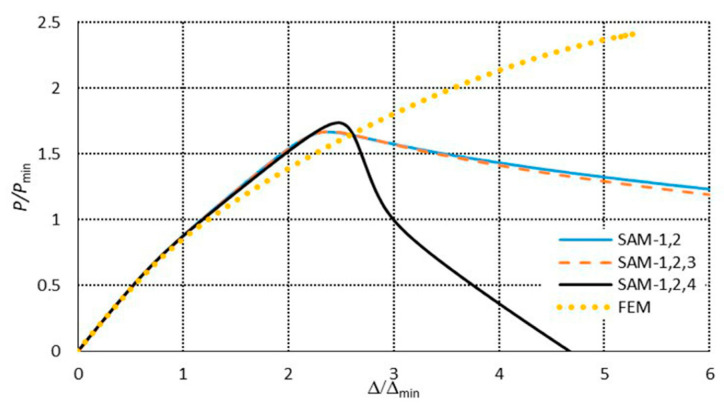
Post-buckling equilibrium paths in the dimensionless system for the 300 mm-long TH-section.

**Figure 16:**
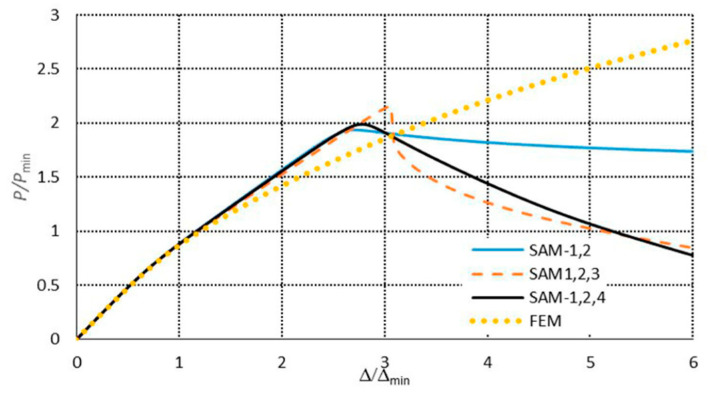
Post-buckling equilibrium paths in the dimensionless system for the 500 mm-long TH-section.

The SAM and FEM calculations for the post-buckling states were conducted analogously as for the C-channels, that is to say, the same level of mode imperfections and two- and three-mode approaches for the SAM were assumed. The post-buckling paths in the systems *P/P*_min_ = *P/P*_1_ versus the shortening Δ/Δ*_min_ =* Δ/Δ_1_ are presented in Figure 15 for 300 mm and in Figure 16 for 500 mm. For both the lengths, the ultimate load-carrying capacity was obtained both for the two-mode (*J =* 2) and three-mode (*J =* 3) approaches. The curves SAM-1,2, SAM-1,2,3, and SAM-1,2,4 overlap up to the ultimate point, and then they fall, but at 300 mm, the SAM-1,2,4 (Figure 15) falls most dramatically. For the length of 500 mm, the curves SAM-1,2,3 and SAM-1,2,4 fall much more sharply than SAM-1,2. For 300 mm (Figure 15), the ultimate point occurs at *P/P*_min_ ≈ 1.7 and Δ/Δ_min_ ≈ 2.25, whereas for 500 mm (Figure 16), it occurs at *P/P*_min_ ≈ 2 and Δ/Δ_min_ ≈ 2.6. The FEM paths obtained for 300 (Figure 15) and 500 mm (Figure 16) are close to the ultimate load-carrying capacity for the SAM, which occurs at *P/P*_min_ > 2.5 and Δ/Δ*_min_* > 5.4. Similarly, as for the C-channels, the FEM computations were catastrophically disrupted due to a lack of numerical convergence.

**Figure 17:**
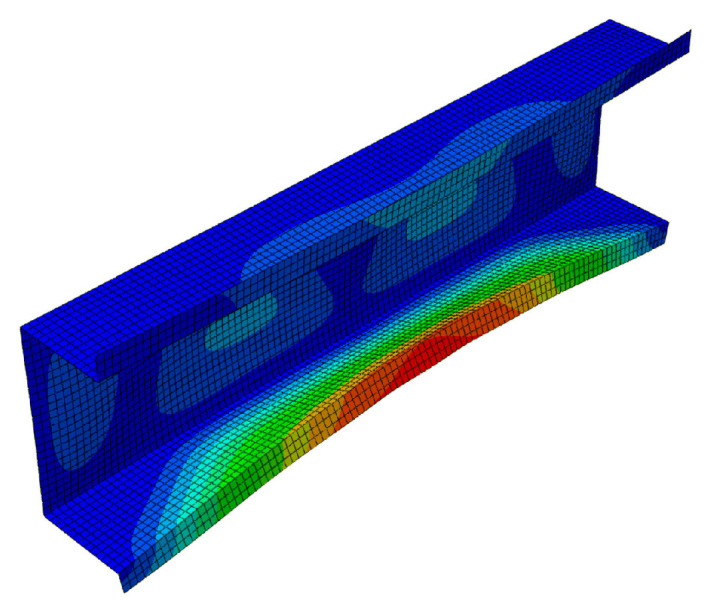
Mode of the 300 mm-long TH-channel column in the ultimate state, FEM results.

**Figure 18:**
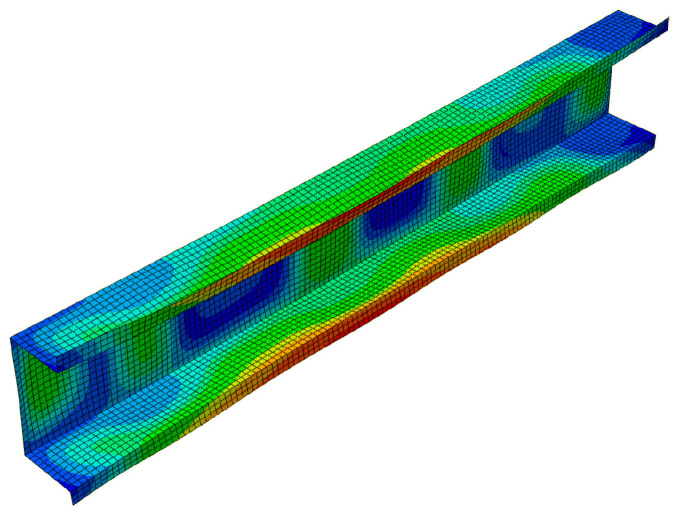
Mode of the 500 mm-long TH-channel column in the ultimate state, FEM results.

In Figure 17 and Figure 18, buckling modes for the FEM ultimate points at 300 and 500 mm, respectively, are presented. The mode for 300 mm (Figure 17) is more asymmetrical than for the 500 mm C-channel (Figure 10). On the other hand, the mode for 500 mm (Figure 18) is symmetrical in practice.

In the FEM, the modes considered in the analysis are not under control as opposed to the SAM. The asymmetrical mode (Figure 17) could be attained via an automatic introduction of anti-symmetrical eigenvalues (i.e., bifurcation ones) into the computations. Edge reinforcements for the top-hat channels result in higher load-carrying capacity when compared to the C-channels.

In the SAM calculations for C- and TH-channels for the SAM-1,2,4 three-mode approach, an unexpected effect of the third global distortional-flexural mode on the interactive buckling and the load-carrying capacity was found. It was not possible to verify the SAM calculations within the FEM; nevertheless, they unexpectedly cast light on a catastrophic disruption of the computations for post-buckling paths due to a lack of convergence, although Rik’s procedure with various initial parameters was applied. The observed numerical phenomena for the SAM and the FEM thus require further intensive investigations with respect to modeling and the methods applied.

## 4. Conclusions

Multimodal buckling of the C- and TH-section (the so-called “top-hat”) columns, made of isotropic material and subjected to compression, was considered. The column lengths were selected to observe strong interactions between various eigenvalues. The problem of nonlinear stability was solved with two methods, namely the semi-analytical method (SAM) using Koiter’s perturbation approach and the finite element method (FEM). The results discussed prove that there is a strong effect of higher global distortional-flexural buckling modes (i.e., secondary and third) on the interactive buckling of C- and TH-columns and their load-carrying capacity. On the post-buckling equilibrium paths prepared within the SAM in the case of an interaction of the lowest local and other modes, when higher global distortional-flexural modes are neglected, the curves grow monotonically. However, if the global distortional flexural buckling mode is considered, the post-buckling equilibrium path grows up to the ultimate point, and then, it falls. This regularity repeats in all the columns under analysis. Moreover, in both the methods, attention is drawn to their conditions, particularly to the FEM catastrophic numerical behavior in all the cases of computations under discussion. Hence, the observed phenomena require further thorough and complex investigations, including the experimental ones. Finally, it should be added that all the considerations presented here refer to the elastic state. Thus, all plastic effects and plastic destruction mechanisms that occurred have been neglected.

## Figures and Tables

**Table 1:** Bifurcation stresses in MPa for the C-channel (Figure 1a) and TH-channel (Figure 1b) columns under analysis; the semi-analytical method (SAM) and finite element method (FEM) results.

Length (mm)	SAM				FEM			
	Mode1	Mode2	Mode3	Mode4	Mode1	Mode2	Mode3	Mode4
**C-channel** (Figure 1a)								
300	74.1 (3)	157	1168	5324	73.3	156	1155	5292
500	74.1 (5)	338	1150	5977	73.5	337	1140	6018
**TH-channel** (Figure 1b)								
300	134.0 (5)	265	986	6355	137.8	268	973	6280
500	133.5 (8)	288	1234	5724	137.5	290	1223	5633
